# Multiple cerebral infarctions associated with lung cancer-induced hypereosinophilia: a case report

**DOI:** 10.1186/s12883-021-02423-1

**Published:** 2021-10-13

**Authors:** Maki Ozaki, Tomoo Mano, Nobuyuki Eura, Kazuhide Horimoto, Masato Takano, Chiho Ohbayashi, Kazuma Sugie

**Affiliations:** 1 Department of Neurology, Nara Medical University School of Medicine Graduate School of Medicine, 840 Shijo-Cho, Kashihara, Nara, 634-8522 Japan; 2 Second Department of Internal Medicine, Nara Medical University School of Medicine Graduate School of Medicine, 840 Shijo-Cho, Kashihara, Nara, 634-8522 Japan; 3 Department of Diagnostic Pathology, Nara Medical University School of Medicine Graduate School of Medicine, 840 Shijo-Cho, Kashihara, Nara, 634-8522 Japan

**Keywords:** Hypereosinophilia, Lung cancer, Cerebral infarction, Watershed area

## Abstract

**Background:**

Hypereosinophilia (HE) is caused by various conditions, including solid and hematologic tumors. Nonetheless, there exist no reports on cerebral infarctions caused by HE associated with lung cancer metastasis to the bone marrow.

**Case presentation:**

We report a case of a 67-year-old man with multiple cerebral infarctions associated with HE. His white blood cell and eosinophil counts were 38,900/μL and 13,600/μL, respectively, at 4 weeks before admission. During treatment for HE, he presented with dysarthria and walking difficulties. Magnetic resonance imaging of the brain showed multiple small infarcts in regions such as the bilateral cortex, watershed area, and cerebellum. Chest computed tomography showed small nodes in the lung and enlargement of the left hilar lymph nodes. Bronchoscopic biopsy did not reveal a tumor; however, bone marrow biopsy showed infiltration of tumor cells. We considered a diagnosis of lung cancer metastasizing to the bone marrow, which induced HE and later caused cerebral infarctions.

**Conclusions:**

This case report demonstrates that metastatic cancer in the bone marrow can induce HE, which can consequently cause multiple cerebral infarctions. Clinicians should consider HE as a cause of multiple cerebral infarctions in patients with cancer.

## Background

Hypereosinophilic syndrome (HES) is a myeloproliferative disorder characterized by a markedly elevated eosinophil count and is associated with the dysfunction of multiple organs. Secondary HES derives from various conditions such as autoimmune diseases, infections, solid cancer, and leukemia [1]. Hypereosinophilia (HE) is defined as an absolute eosinophil count of > 1.5 × 10^9^/L on two examinations separated by at least 1 month and/or pathological confirmation of tissue HE. Organ damage-induced HE is defined as HES. Several reports on HE induced by solid and hematologic cancers such as paraneoplastic syndrome have been documented; nonetheless, it remains a rare condition. One of the mechanisms of HE is the production of several cytokines, including interleukin-3, interleukin-5, and granulocyte-macrophage colony-stimulating factor (GM-CSF), by the primary cancer, which accounts for the increased production of eosinophilic granulocytes in the bone marrow. Another mechanism is an eosinophilotactic response due to necrosis in the tumor and increased production of eosinophils due to tumor cell dissemination in the bone marrow [2]. However, cases in which cancer bone metastasis itself induces HE are rare. Regardless of the cause, several patients with HE develop cerebral infarction, particularly in the watershed area. We report a case of a watershed area infarction due to HE caused by lung cancer metastasis to the bone marrow.

## Case presentation

A 67-year-old man with type 2 diabetes mellitus presented to our hospital with an elevated white blood cell (WBC) count (38,900/μL) and eosinophil count (13,600/μL). He had no allergies or showed signs of infection. Malignancy was suspected, and some tumor markers were examined on the same day, including carcinoembryonic antigen (CEA), carbohydrate antigen 19–9 (CA19–9), neuron-specific enolase (NSE), progastrin-releasing peptide (ProGRP), squamous cell carcinoma (SCC), cytokeratin fragment (CYFRA), sialyl Lewis X-i antigen (SLX), and soluble interleukin-2 receptor (sIL-2R). sIL-2R concentration was remarkably elevated at 3421 IU/mL. To identify the cause of these hematologic abnormalities, chest computed tomography (CT) was performed, which revealed small nodes on both sides of the lungs and enlargement of the left hilar and right subclavian lymph nodes. Lung cancer metastasis to the bone marrow was suspected; thus, bronchoscopic biopsy and bone marrow biopsy were performed.

Four weeks later, he noticed weakness on both sides of his arms. He could not walk and speak well on the next day. Subsequently, he was admitted to our hospital at 2 days after the appearance of the first symptoms. On admission, the patient was 167 cm tall, and his body weight was 53.3 kg. On examination, his blood pressure was 126/72 mmHg, his pulse was 79 beats per minute and regular, and his temperature was 36.9 °C. His consciousness level was E4V4M6 on the Glasgow Coma Scale. Impaired attention and visuospatial cognition, simultanagnosia, and oculomotor apraxia were observed [3]. He presented with dysarthria and mild limb weakness. Barré and Mingazzini signs were positive bilaterally. Deep tendon reflexes were accelerated on both sides of the upper and lower limbs, and Babinski’s sign was also positive. It was not possible to assess sensory, proprioceptive, joint, or vibration changes.

Peripheral blood examinations revealed leukocytosis with HE. His WBC count was elevated to 71,500/μL and eosinophil count was 36,465/μL, accounting for 51.0% of WBC. Finding for other inflammatory markers including anti-nuclear antibody, myeloperoxidase-antineutrophil cytoplasmic antibody (MPO-ANCA), proteinase 3-ANCA (PR3-ANCA), anti-cardiolipin antibody, and lupus anticoagulant were negative. D-dimer level was 1.2 μg/mL. Antithrombin III activity, prothrombin time-international normalized ratio (PT-INR), activated partial thromboplastin time (APTT), and protein C and protein S activities were normal. Hemoglobin A1c (HbA1c) level was 8.4%. Findings related to tumor makers were as follows: the sIL-2R level was 4212 IU/mL and the levels of other tumor makers (NSE, CYFRA, SLX) were slightly elevated. Brain natriuretic peptide (BNP) level was slightly elevated at 47.5 pg/mL.

**Fig. 1:**
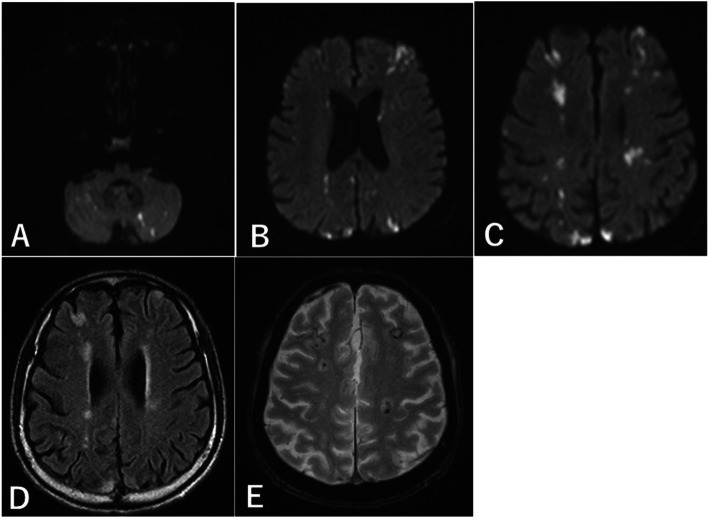
MRI images. **a-c** Axial diffusion-weighted image and **d** fluid-attenuated inversion recovery of MRI show infarctions in the bilateral watershed area of the middle cerebral artery and the cerebellar hemispheres. **e** Follow-up susceptibility-weighted imaging on day 15 shows the increasing microbleeds in infarcts. MRI, magnetic resonance imaging

Magnetic resonance imaging (MRI) of the brain showed multiple acute small infarcts in the bilateral cortex, watershed area of the middle cerebral artery, and cerebellar hemispheres (Fig. 1a-d). Some of them showed hemorrhagic infarction. Magnetic resonance angiography (MRA) of the head and neck showed normal cerebral vasculature. Ultrasound examination of the carotid arteries indicated no stenosis or low echoic plaques. Holter electrocardiography did not detect any arrhythmia. Transthoracic echocardiography showed partial thickening of the left ventricular wall. The left ventricular ejection fraction was 68%, and no valvular abnormality was revealed.

**Fig. 2:**
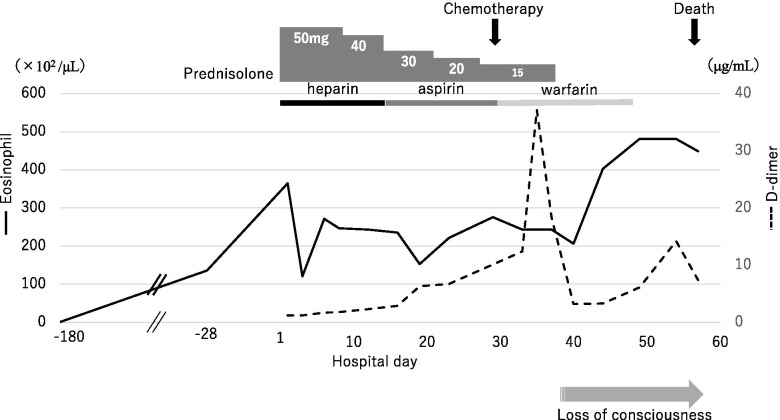
Timeline. A timeline of the changes in eosinophil count and D-dimer levels with treatment is provided. After treatment was initiated with prednisolone for hypereosinophilia and unfractionated heparin for acute cerebral infarction, the eosinophil count was slightly decreased. Antithrombotic therapy was changed from heparin to aspirin preciously, but the D-dimer level increased; therefore, it was changed to warfarin. Despite the chemotherapy and steroid therapy, his eosinophil count continued to increase, and his blood results indicated disseminated intravascular coagulation. Consequently, he died on day 57 of admission

On admission day, 2 days had already passed after the appearance of the first symptoms of infarction, and we could not carry out acute reperfusion therapy. Treatment was initiated with 50 mg/day of prednisolone for HE and 5000 units/day of unfractionated heparin for acute cerebral infarction (Fig. 2). We gradually increased the heparin dose to 8000 units/day to avoid APTT overextension. His eosinophil count slightly decreased, and simultanagnosia and oculomotor apraxia disappeared. Follow-up MRI of the brain on day 15 of admission showed no increase in infarcts; however, microbleeds in the infarcts were increased (Fig. 1e). We changed heparin to aspirin in consideration of hemorrhage.

**Fig. 3:**
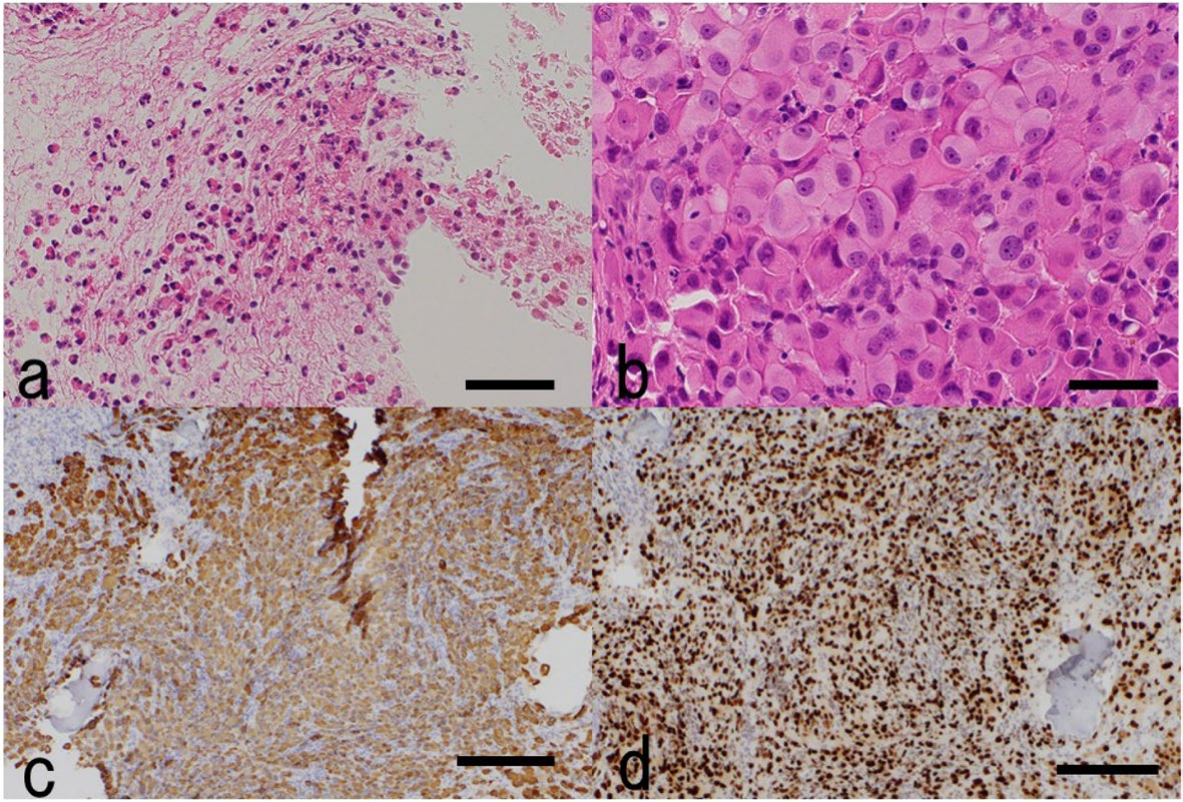
Pathological images. Bronchoscopic biopsies revealed eosinophilic infiltration (**a**: hematoxylin and eosin). Bone marrow biopsies revealed infiltration of cancer cells, eosinophils, and neutrophils (**b**: hematoxylin and eosin). Immunostaining suggests lung adenocarcinoma (**c**: cytokeratin 7, **d**: thyroid transcription factor-1). Bar = 50 μm (**a**, **b**), 200 μm (**c**, **d**)

On day 22 of admission, the pre-admission bronchoscopic biopsies revealed eosinophilic infiltration of the bronchial epithelium and infiltration of cancer cells, eosinophils, and neutrophils into the bone marrow. Immunostaining of a bone marrow sample revealed the following pattern suggestive of lung adenocarcinoma: cytokeratin 7(+), cytokeratin 20(−), and thyroid transcription factor-1(+) (Fig. 3). He was diagnosed with clinical stage IVb lung adenocarcinoma.

We considered that the cerebral infarctions were caused by HE induced by lung cancer metastasis to the bone marrow. As warfarin is not appropriate for drug interaction, we then continued treatment with aspirin. On day 30, chemotherapy with pemetrexed for adenocarcinoma was initiated. We subsequently discovered that the elevation in D-dimer level was associated with deep vein thrombosis; hence, we changed the antithrombotic drug to warfarin. Despite chemotherapy and steroid therapy, the number of eosinophils did not decrease. Therefore, we tapered and then stopped the use of prednisolone.

After the first round of chemotherapy, his WBC and eosinophil counts continued to increase, and his blood results indicated disseminated intravascular coagulation (DIC). His general condition deteriorated, and he died on day 57.

## Discussion and conclusions

This case highlights a rare case of multiple cerebral infarctions associated with lung cancer metastasis-induced HE. There are several case reports on multiple cerebral infarctions due to ANCA-associated vasculitis [4–7]. Most of these cases presented with other clinical features (asthma, sinusitis, and skin rash) and were ANCA-positive. However, we did find a case of cerebral infarctions associated with ANCA-negative eosinophilic granulomatosis with polyangiitis (EGPA) who had no clinical features [7]. This case was found to have granulomas with epithelial cells, eosinophilic infiltration, and vasculitis on autopsy. In our case, small nodes in the lung revealed on chest CT and a high serum sIL-2R level were suggestive of HE induced by a tumor rather than ANCA-associated vasculitis. After admission, the bone marrow biopsy revealed infiltration of tumor cells, and the patient had no clinical features of ANCA-associated vasculitis or other causes of HE. Therefore, we considered that the lung cancer metastasis to the bone marrow induced HE. We continued to administer therapy but did not add immunosuppressant agents such as rituximab and omalizumab.

There are several reports on HE caused by solid or hematologic tumors metastasizing to the bone marrow. Table 1 shows the reported cases of cancer metastasis to the bone marrow presenting with HE. The mechanism of HE development in association with cancer is hypothesized to be a paraneoplastic leukemoid reaction that stimulates the bone marrow via the production of tumor-produced cytokines, interleukin-5, and GM-CSF [8]. However, there is another hypothesis that an eosinophilotactic response occurs through necrosis of tumor cells disseminated in the bone marrow [2]. Systemic therapy, including glucocorticoids, hydroxyurea, and vincristine, is effective in reducing peripheral eosinophilic counts in paraneoplastic eosinophilia. However, in our case, neither steroid administration nor chemotherapy alleviated HE, and this became particularly evident in the biopsy results. Thus, we considered that the mechanism underlying HE development in this patient was not paraneoplastic; rather, it was eosinophilotactic. However, the exact mechanism of HE development in this patient could not be concluded.

**Table 1:** Reported cases of hypereosinophilia secondary to cancer metastasis to the bone marrow

	Age	Primary cancer	Bone metastasis	Eosinophils (/μL)	Cerebral infarction
Todenhöfer et al., 2012 [2]	46	RCC	+	21,636	+
Akkad et al., 2020 [8]	68	NSCLC	+	79,560	+
Verstraeten et al., 2011 [9]	65	NSCLC	+	41,040	–
Evangeline et al., 2006 [10]	72	Thyroid carcinoma	+	1600	–
Our case	67	NSCLC	+	47,481	+

*NSCLC* non-small cell lung carcinoma, *RCC* renal cell carcinoma

Wu et al. reported that approximately 12% of patients with HES have cerebral infarctions [11]. Almost all cases of HE-induced cerebral infarctions were in the watershed area [12, 13], and our case is no exception. Infarctions in the watershed area are usually caused by hemodynamic mechanisms such as internal carotid artery stenosis or severe cardiac dysfunction. However, HE itself can induce an infarction in the watershed area. Therefore, MRI, particularly MRA, is very important for the examination of the etiology of watershed area infarctions [14].

Aida et al. reported that cerebral perfusion is lower in the watershed area than in the other areas of the brain. HE increases the consistency of blood and impairs clearance in this area [15]. Sarazin et al. reported that HE induces cardiomyopathy and causes microembolisms in the watershed areas [16]. In our case, we did not perform myocardial biopsy, but partial posterior left ventricular wall thickening was found on transthoracic echocardiography. This may be due to eosinophilic myocarditis, and we suspect that cardiac microthrombi may have caused microembolisms, which were not cleared in the watershed area.

According to the 2021 American Heart Association stroke guidelines, the potential beneficial effect of heparin on stroke prevention is unknown [17]. There exists no established medication treatment for cerebral infarctions in HE. However, empirically, antiplatelets and anticoagulants, including heparin, are frequently used because its etiology is thought to be both a hyperviscous state and microembolisms [12, 18]. Hence, we first used unfractionated heparin with steroid therapy.

In conclusion, we reported a case of multiple cerebral infarctions in the watershed area caused by cancer-induced HE. HE can sometimes cause cerebral infarction; however, there are few reports on cerebral infarction with HE caused by metastatic cancer in the bone marrow. HE should be considered as a cause of multiple cerebral infarctions in patients with cancer.

## Data Availability

Not applicable.

## Abbreviations

ANCA: Antineutrophil cytoplasmic antibody
APTT: Activated partial thromboplastin time
BNP: Brain natriuretic peptide
CT: Computed tomography
CYFRA: Cytokeratin fragment
DIC: Disseminated intravascular coagulation
GM-CSF: Granulocyte-macrophage colony-stimulating factor
HbA1c: Hemoglobin A1c
HE: Hypereosinophilia
HES: Hypereosinophilic syndrome
MRA: Magnetic resonance angiography
MRI: Magnetic resonance imaging
MPO-ANCA: Myeloperoxidase-antineutrophil cytoplasmic antibody
NSE: Neuron specific enolase
PR3-ANCA: Proteinase 3- antineutrophil cytoplasmic antibody
PT-INR: Prothrombin time-international normalized ratio
sIL-2R: Soluble interleukin-2 receptor
SLX: Sialyl Lewis X-i antigen
WBC: White blood cell
